# Impulsive decision-making predicts the course of substance-related and addictive disorders

**DOI:** 10.1007/s00213-020-05567-z

**Published:** 2020-06-05

**Authors:** Anja Kräplin, Michael Höfler, Shakoor Pooseh, Max Wolff, Klaus-Martin Krönke, Thomas Goschke, Gerhard Bühringer, Michael N. Smolka

**Affiliations:** 1 Faculty of Psychology, Technische Universität Dresden, Dresden, Germany; 2 Work Group Addictive Behaviors, Risk Analysis and Risk Management, Faculty of Psychology, Technische Universität Dresden, Chemnitzer Straße 46, 01187 Dresden, Germany; 3 Department of Psychiatry, Technische Universität Dresden, Dresden, Germany; 4 Freiburg Center for Data Analysis and Modeling, University of Freiburg, Freiburg im Breisgau, Germany; 5 IFT Institut für Therapieforschung, Munich, Germany; 6 Department of Clinical Research, Faculty of Health, University of Southern Denmark, Odense, Denmark

**Keywords:** Substance use disorders, Behavioural addictions, Decision-making, Impulsivity, Risk-seeking, Loss aversion

## Abstract

**Background:**

This study investigated whether patterns of impulsive decision-making (i) differ between individuals with DSM-5 substance use disorders (SUD) or non-substance-related addictive disorders (ND) and healthy controls, and (ii) predict the increase of SUD and ND severity after one year.

**Methods:**

In a prospective-longitudinal community study, 338 individuals (19–27 years, 59% female) were included in one of three groups: SUD (*n* = 100), ND (*n* = 118), or healthy controls (*n* = 120). Group differences in four impulsive decision-making facets were analyzed with the Bayesian priors: delay discounting (mean = 0.37, variance = 0.02), probability discounting for gains and for losses (each − 0.16, 0.02), and loss aversion (− 0.44, 0.02). SUD and ND severity were assessed at baseline and after 1 year (*n* = 312, 92%). Predictive associations between decision-making and SUD/ND severity changes were analyzed with the Bayesian prior: mean = 0.25, variance = 0.016.

**Results:**

Compared with controls, the SUD group displayed steeper delay discounting and lower probability discounting for losses; the ND group displayed lower probability discounting for losses (posterior probabilities > 98%). SUD symptom increase after 1 year was predicted by steeper delay discounting and lower loss aversion; ND symptom increase by lower probability discounting for losses and lower loss aversion (posterior probabilities > 98%). There was low evidence for predictive relations between decision-making and the quantity-frequency of addictive behaviours.

**Discussion:**

Impulsive decision-making characterizes SUD and ND and predicts the course of SUD and ND symptoms but not the engagement in addictive behaviours. Strength of evidence differed between different facets of impulsive decision-making and was mostly weaker than a priori expected.

**Electronic supplementary material:**

The online version of this article (10.1007/s00213-020-05567-z) contains supplementary material, which is available to authorized users.

## Introduction

Addictive disorders (AD) are paradigmatic examples of impulsive choice behaviour. Individuals with AD prefer highly probable, immediate outcomes, such as hedonic experiences or to avoiding stress, each at the expense of possible long-term health, social, and financial benefits. Consequently, several AD models have focused on dysfunctional decision processes as underlying neurocognitive mechanisms of AD (for an overview, see Goschke 2014; Redish et al. 2008). In this study, we aimed to address the important open research questions of whether different facets of impulsive decision-making (i) differentiate between individuals with substance use disorders (SUD) or non-substance-related addictive disorders (ND) and healthy controls and (ii) predict the increase of AD severity.

A first identified need for research is to better characterize patterns of impulsive choices in AD (Busemeyer and Stout 2002; Diekhof et al. 2008; Fellows and Farah 2005; Yechiam et al. 2005). There is no uniform definition of impulsive decision-making. In choices with two known options, it is important to consider whether the options concern delayed or uncertain outcomes (Shead and Hodgins 2009) and whether the outcome is a gain or a loss (Ohmura et al. 2005). It was argued for a more nuanced view of impulsive decision-making, i.e. that it is important to measure multiple facets of impulsivity in order to better understand different aspects of decision-making (Green and Myerson 2013). Based on this previous research, impulsive decision-making in AD should be defined by several facets that reflect the well-known AD-related changes in cognitive control and reward and punishment processing (Goschke 2014): a lower ability to delay gratification (e.g. smoking for immediate reward), an increased risk-taking propensity for uncertain rewards (e.g. betting wins again instead of keeping the winnings), and a reduced sensitivity to potential losses (e.g. worse grades due to excessive alcohol consumption). Previous studies have mostly focused on the ability to delay gratification, which is typically measured by delay discounting tasks. Researchers found consistent evidence that individuals with SUD or ND are characterized by steeper delay discounting (for an overview, see Amlung et al. 2017; Bickel et al. 2014; Kluwe-Schiavon et al. 2020). As another facet of impulsive decision-making, some studies used probability discounting tasks to assess the risk-taking propensity either for uncertain rewards (probability discounting for gains) or to avoid certain losses (probability discounting for losses). Other studies used mixed gambles tasks to assess loss aversion, i.e. the tendency to weight the absolute value of losses higher as the absolute value of gains. We hypothesized that AD is characterized by more impulsive decision-making within these facets compared with healthy controls, which would be indicated by lower probability discounting for gains, lower probability discounting for losses, and lower loss aversion. While some studies found the assumed group differences between samples with SUD (Bernhardt et al. 2017; Brevers et al. 2014) or ND and healthy controls (Brevers et al. 2012; Holt et al. 2003; Li et al. 2016; Lorains et al. 2014; Madden et al. 2009), there were also studies that found no or opposite evidence in SUD (Mejía-Cruz et al. 2016; Ohmura et al. 2005; Reynolds et al. 2004; Takahashi et al. 2009) and ND (Gelskov et al. 2016; Giorgetta et al. 2014; Takeuchi et al. 2015). Previous studies mostly used one decision-making task and found mixed evidence. To address the open question whether all or certain facets of impulsive decision-making are affected in AD requires a common experimental and analytical approach (Green and Myerson 2004). Previous research from our lab has examined the four facets of impulsive decision-making presented above within a common approach (Pooseh et al. 2018). It was shown that patients with alcohol use disorder displayed a steeper delay discounting, lower probability discounting for gains, lower probability discounting for losses, and lower loss aversion compared with healthy controls (Bernhardt et al. 2017). It is important to further study different impulsive decision-making facets in AD to understand the role of changes in the different underlying processes for the onset and course of AD. For example, steeper delay discounting may underlie the initiation of substance use (as immediate substance effects are overvalued despite negative future consequences for health) while lower probability discounting for gains may underlie a steeper progression of AD (as individuals prefer the chance of more rewarding effects of addictive behaviours to the less rewarding effects of other reinforcers such as leisure activities). The precise mapping of behavioural differences in these multiple decision-making facets is a prerequisite for discovering the multiple underlying brain mechanisms and supports research on transdiagnostic processes (Green and Myerson 2013). For example, literature reviews and meta-analyses concerning delay discounting consistently showed a steeper delay discounting in a number of mental disorders such as SUD, bipolar disorders, personality disorders, or depressive disorders (Amlung et al. 2019; Bickel et al. 2019; Bickel et al. 2012). It was therefore argued that steeper delay discounting accounts for common symptoms across diagnostic categories and acts as a transdiagnostic process (Amlung et al. 2019; Bickel et al. 2019; Bickel et al. 2012), which is based on an imbalance between two competing neurobehavioural decision systems (Bickel Warren and Yi 2008). For probability discounting and loss aversion, more empirical evidence is needed, which was one main motivation to conduct this study. As a first step to address this research need, impulsive decision-making patterns were compared between individuals with SUD or ND and healthy controls. This deepens our understanding of common and different underlying mechanisms (Petry et al. 2014; Shaffer et al. 2004) and facilitates the detection of further transdiagnostic processes related to maladaptive decision-making (Bickel et al. 2019).

A second identified need for research is to determine whether different facets of impulsive decision-making *predict the course* of AD severity, which requires a longitudinal design (Gowin et al. 2018). Up to now, a small number of longitudinal studies have reported small to medium predictive associations between steeper delay discounting and an increased likelihood of smoking initiation (for an overview, see Barlow et al. 2017) or later alcohol involvement in healthy adolescents (Fernie et al. 2013). However, there was only weak evidence that impulsive decision-making predicts the alcohol quantity-frequency index (QFI) 1 year later (Bernhardt et al. 2017) or alcohol use, intoxication, and problems 2 years later (Fernández-Artamendi et al. 2018). Concerning longitudinal evidence, three research needs are addressed in this study. First, there is a lack of longitudinal studies that focus on other facets of impulsive decision-making than delay discounting. It remains unclear whether other facets of impulsive decision-making, such as lower loss aversion, are also or even more relevant for predicting AD severity. Exploring this is of central importance, since decisions in AD do not only include immediate and delayed reward options but also risks and negative consequences (e.g. health problems). Second, there is a lack of longitudinal studies that investigate multiple AD with and without substance use. For example, the predictive relationships between delay discounting and tobacco use disorder may be stronger than for computer use disorder, as a steeper delay discounting also occurs as a consequence of substance use, which is not relevant for behavioural addictions (De Wit 2009; Weafer et al. 2014). Third, there is a lack of longitudinal studies on the course of AD that distinguish between clinical aspects (symptoms) and engagement in addictive behaviours (quantity and frequency). Therefore, clear conclusions about which aspect impulsive decision-making is predictive for remain unclear.

To address the identified research needs, we first compared different facets of impulsive decision-making between individuals with SUD or ND and healthy controls. Second, we applied a longitudinal design to analyze predictive associations between different facets of impulsive decision-making and SUD or ND severity 1 year after. We used a Bayesian approach to analyze evidence for the hypotheses that steeper delay discounting, lower probability discounting for gains, lower probability discounting for losses, and lower loss aversion (i) characterize individuals with SUD or ND by varying degrees compared with healthy controls and (ii) predict an increase in the course of SUD or ND severity, defined as number of fulfilled diagnostic criteria and QFI. The Bayesian approach has several advantages over the critically debated null hypothesis significance testing, e.g. (a) prior assumptions about parameters of interest need to be explicitly stated (instead of implicitly assuming that all were equally likely), (b) existing knowledge and data from previous studies can be included, and (c) the posterior probabilities provide the probability that a tested hypothesis is true (for a general overview, see Baldwin and Larson 2017; for an overview within the topic delay discounting, see Franck et al. 2019).

## Methods

### Design and procedure

Data were collected as part of a prospective-longitudinal community study within a Collaborative Research Centre (SFB 940) at the Technische Universität Dresden, Germany. At baseline, participants took part in four different test sessions including a clinical assessment, a behavioural task battery, an fMRI session, and experience sampling for daily self-control failures (for further results, see Krönke et al. 2018; Krönke et al. 2020; Wolff et al. 2016). The 1-year follow-up included only a clinical assessment. According to the focus of these analyses, the two clinical assessments and the decision-making tasks will be described in detail.

### Recruitment and participants

**Fig. 1:**
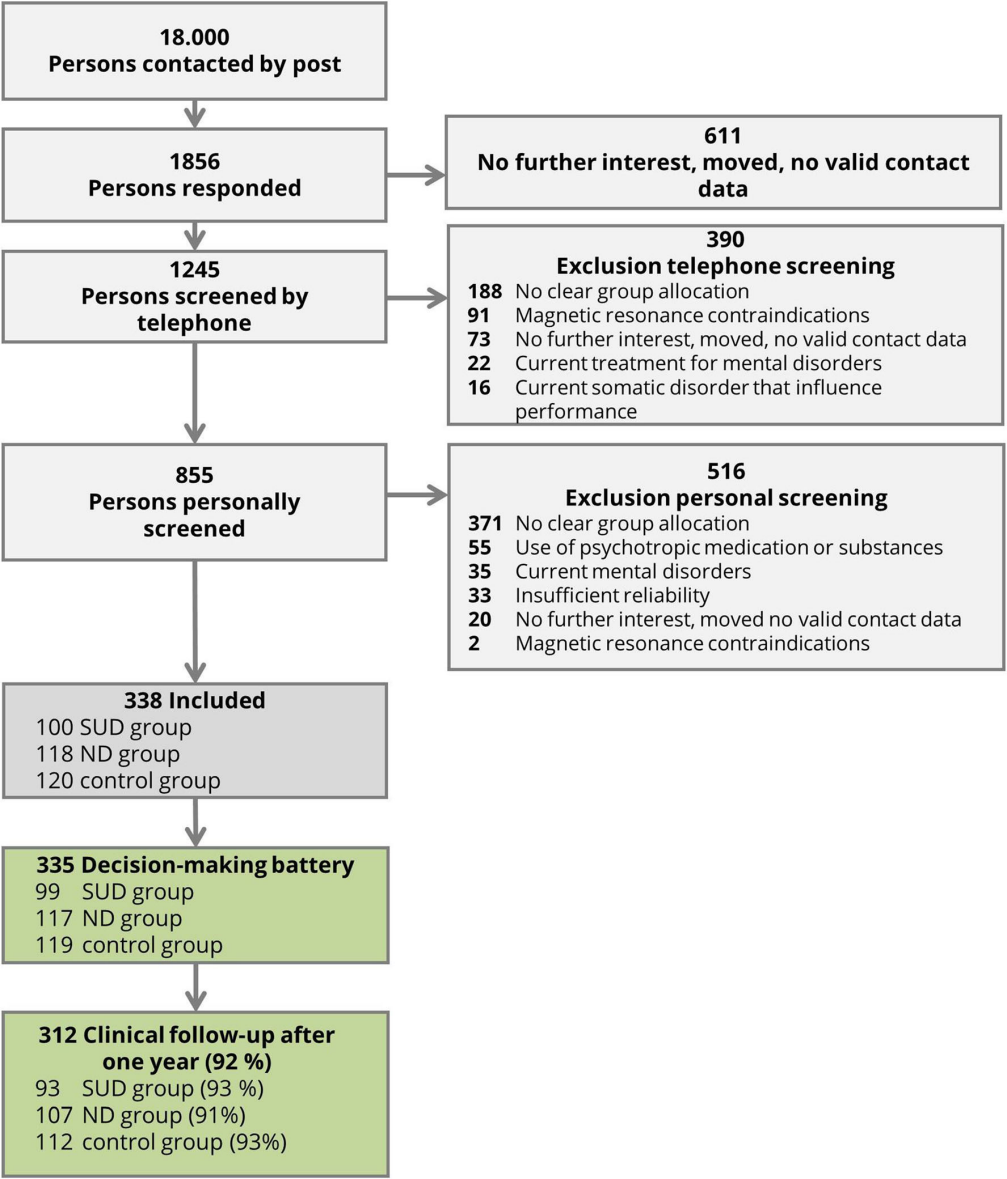
Participant flowchart with numbers and reasons of inclusion and exclusion

From 2013 to 2016, 18,000 inhabitants aged between 19 and 27 randomly taken from the registration office files of Dresden were invited by post to participate in our study and 1856 responded to the invitation letter (10.3 %, see Fig. 1; for comparison of respondents and non-respondents, see Table S1 in the supplemental material). The community sample and the age range were chosen as the best balance between minimal interference from neurodevelopmental processes (Casey and Jones 2010), maximum changes in AD symptoms (Wagner and Anthony 2002; Wittchen et al. 2008), and minimal interferences with task performance due to neurological damages caused by long-term substance use in patient samples (often recruited within previous studies) (Naim-Feil et al. 2013). Included participants had to fulfil the criteria for one of three groups: In the SUD group, participants had a diagnosis of alcohol and/or tobacco use disorder according to the fifth edition of the Diagnostic and Statistical Manual of Mental Disorders (DSM-5; American Psychiatric Association (APA) 2013) but no lifetime ND. In the ND group, participants were included who fulfilled two or more criteria for a DSM-5 gambling disorder or for an AD related to Internet use (not for gambling, gaming, or shopping), gaming, or shopping assessed with adapted criteria from DSM-5 SUD. Participants in the ND group had no lifetime SUD. The control participants had no current or lifetime SUD or ND. Exclusion criteria for all participants were (1) no written informed consent or limited ability to understand the questionnaires and tasks, (2) disorders that might influence cognition or motor performance (e.g. craniocerebral injury), (3) magnetic resonance contraindications, (4) current treatment for mental disorders, or (5) use of psychotropic medication or substances. Applying the inclusion and exclusion criteria, 855 participants were invited for a personal diagnostic screening. In the personal screening, we used the Munich-Composite International Diagnostic Interview (DIA-X/M-CIDI; Wittchen and Pfister 1997) to assess the following further exclusion criteria: (6) lifetime psychotic symptoms, bipolar disorder, and other SUD or ND not under study, and (7) major depression, somatoform, anxiety, obsessive compulsive, or eating disorders within the last 4 weeks. Finally, 338 participants were included in the study (Table 1; see supplemental material for sample size calculation). After 1 year, 312 participants were contacted again (retention rate: 92%) to assess AD criteria and QFI with a standardized clinical interview (see Table S2 in the supplemental material for follow-up socio-demographic data). All participants gave their written informed consent and the study protocol was approved by the Institutional Review Board at the Technische Universität Dresden (EK45022012).

**Table 1:** Demographic characteristics of the baseline sample (*n* = 338) separately for the substance-related disorder (SUD) group, the non-substance-related addictive disorder (ND) group, and the control group.

	SUD	ND	Controls
	100	118	120
	*M* (SD)	*M* (SD)	*M* (SD)
Age	21.8 (1.6)	21.8 (1.7)	21.9 (1.8)
Intelligence quotient	103.7 (8.9)	104.4 (10.1)	104.8 (10.4)
	*n* (%)	*n* (%)	*n* (%)
Female participants	53 (53.0)	70 (59.3)	76 (63.3)
Income < 1500 Euro per month	75 (75.8)	92 (77.0)	89 (75.4)
School qualification ‘Abitur’^1^	68 (68.7)	85 (72.0)	97 (82.2)
In education, pupil, or student	72 (72.7)	87 (73.7)	87 (72.57)^2^

*M*, means; *SD*, standard deviations

^1^Abitur is the German school-leaving qualification required for university entrance

^2^Two participants refused to provide information

### Measurements

#### Addictive disorder groups and severity at baseline

In the personal diagnostic session, participants were interviewed with a modified version of the DIA-X/M-CIDI (Wittchen and Pfister 1997). The standardized diagnostic interviews were carried out by advanced students of psychology who were trained in clinical assessments and supervised by the first author. Within the interviews, the quantity and frequency of the behaviours of interest in the last 12 months were first asked (e.g. “In the last 12 months, how many hours did you surf the Internet on a normal day?”). Then, it was evaluated for each behaviour whether the criteria for DSM-5 SUD or the criteria for ND adapted according to DSM-5 SUD were met (e.g. “Have you ever tried unsuccessfully to limit the use of the Internet for a few days?”). While DSM-5 defines SUD as fulfilment of 2 or more out of 11 criteria, ND has a higher diagnostic threshold for historical but not empirical reasons. This might be one explanation why previous research has suggested that socio-demographic and addiction-related characteristics of individuals with subthreshold ND more closely resemble those of individuals with ND than those of individuals without endorsing any clinical criteria (Cox et al. 2004; Sassen et al. 2011). In order to achieve a homogenous group definition, we also defined ND as fulfilment of 2 or more out of 11 criteria adapted from SUD.

From 100 participants who were allocated to the SUD group, 45 individuals (45%) fulfilled diagnostic criteria for alcohol use disorder, 39 (39%) for tobacco use disorder, and 16 (16%) for both. From 118 participants who were allocated to the ND group, 83 participants (70%) had an ND related to Internet use (i.e. use of social networks, chats, e-mails, forums, info search, entertainment, pornography), 16 (14%) to gaming, 18 (15%) to Internet use and gaming, 1 to gambling (1%), and none to shopping.

#### Changes in addictive disorder severity towards follow-up

The first indicator of AD severity was the number of fulfilled DSM-5 criteria. DSM-5 defines addiction severity according to the specifiers mild, i.e. 2 to 3 criteria, moderate, i.e. 4 to 5 criteria, and severe, i.e. 6 to 11 criteria. As expected for our sampling procedure, the AD severity was mainly mild (baseline 62%; follow-up 67%) and moderate (baseline 28%; follow-up 26%; see Table S3). The dependent variables in our analyses were the *z*-standardized sum of the fulfilled diagnostic criteria at baseline and at follow-up, separately for SUD and ND (*z*-standardization was conducted for a better interpretation of regression coefficients; see Table 2 for unstandardized values).

**Table 2:** Median and range of addictive disorder severity at baseline and 1-year follow-up separately for the substance use disorder (SUD) group, the non-substance-related addictive disorder (ND) group, and the control group

	SUD^1^	ND^1^	Controls
	Median (range)	Median (range)	Median (range)
Baseline			
Sum of DSM-5 SUD criteria	3 (2–13)	0 (0–2)	0 (0–2)
Tobacco-related and/or	2 (0–7)	0 (0–1)	0 (0–1)
Alcohol-related	2 (0–6)	0 (0–1)	0 (0–1)
Sum of (adapted) DSM-5 ND criteria	0 (0-2)	4 (2–15)	0 (0–1)
Internet-related and/or	0 (0–1)	3 (0–9)	0 (0–1)
Gaming-related and/or	0 (0–1)	0 (0–8)	0 (0–1)
Gambling-related and/or	0 (0–1)	0 (0–2)	0 (0–1)
Shopping-related	0 (0–1)	0 (0–1)	0
Quantity frequency indices SUD-related			
Tobacco-related (cigarettes per week) and/or	10 (0–140)	0 (0–35)	0 (0–49)
Alcohol-related (gram alcohol per week)	72 (0–630)	18 (0–360)	22.5 (0–180)
Quantity frequency indices ND-related (hours per week)			
Internet-related and/or	0 (0–42)	14 (0–70)	0 (0–35)
Gaming-related and/or	0 (0–14)	0 (0–35)	0 (0–28)
Gambling-related and/or	0 (0–4)	0 (0–2)	0
Shopping-related	0 (0–6)	0 (0–2)	0
Follow-up (1 year)			
Sum of DSM-5 SUD criteria	2 (0–8)	0 (0–6)	0 (0–6)
Tobacco-related and/or	0 (0–6)	0 (0–6)	0 (0–4)
Alcohol-related	0 (0–5)	0 (0–5)	0 (0–4)
Sum of (adapted) DSM-5 ND criteria	0 (0–6)	1 (0–15)	0 (0–3)
Internet-related and/or	0 (0–6)	2 (0–8)	0 (0–3)
Gaming-related and/or	0 (0–1)	0 (0–8)	0 (0–2)
Gambling-related and/or	0 (0–1)	0 (0–2)	0
Shopping-related	0	0 (0–2)	0 (0–2)
Quantity frequency indices SUD-related			
Tobacco-related (cigarettes per week) and/or	14 (0–140)	0 (0–42)	0 (0–56)
Alcohol-related (gram alcohol per week)	63 (0–540)	27 (0–288)	24.8 (0–360)
Quantity frequency indices ND-related (hours per week)			
Internet-related and/or	12 (0.1–70)	14 (0.5–56)	8 (0–42)
Gaming-related and/or	0.5 (0–35)	0.5 (0–28)	0 (0–35)
Gambling-related and/or	0 (0–2.5)	0 (0–8)	0
Shopping-related	0 (0–1.5)	0 (0–4)	0 (0–14)

^1^According to our inclusion criteria, participants within the addiction groups may fulfil only one or several disorders, e.g. only an alcohol use disorder in the SUD group. Therefore, 0 criteria or a QFI of 0 can also occur within the addiction groups, e.g. if someone has an alcohol use disorder but does not smoke

The second indicator of AD severity was the QFI of use. Quantity was assessed either by standard drinks (alcohol use), cigarettes (tobacco use), or hours of use (Internet use, gaming, gambling, shopping). Frequency was assessed by using the following categories according to the DIA-X/M-CIDI: almost daily, 3–4 times per week, 1–2 times per week, 1–3 times per month, less often than monthly. The QFI was computed by multiplying the maximum of the category and the quantity. As dependent variable, we *z*-standardized the QFIs in long data format to make them comparable over addictive behaviours (gram ethanol, cigarettes, and hours per week) and time (baseline, follow-up), summed up the values separately for substance- and non-substance-related addictive behaviours and *z*-standardized them again for a better interpretation of regression coefficients.

#### Impulsive decision-making

**Fig. 2:**
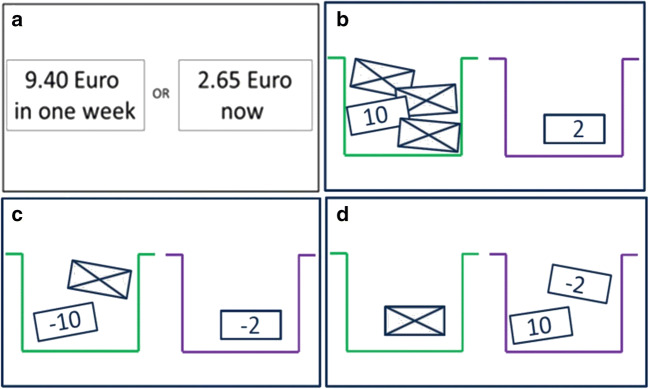
Schematic overview of the tasks in our decision-making battery. **a** Delay discounting task. **b** Probability discounting for gains. **c** Probability discounting for losses. **d** Mixed gambles task

The following four tasks developed by Pooseh et al. (2018; MATLAB scripts available from https://github.com/spooseh/VBDM) were used to assess impulsive decision-making: (1) Delay discounting task with delays of 3, 7, 14, 31, 61, 180, and 365 days between the choice options. Probability discounting tasks (2) for gains and (3) for losses with five possible probability values: 2/3, 1/2, 1/3, 1/4, and 1/5. These three tasks consisted of 30 trials each and monetary gains/losses ranged from 0.30 to 10 €. In the (4) mixed gambles task, participants started with 10 € and played 40 trials with 1–40 € for gains and 5–20 € for losses. In all four tasks (overview in Fig. 2), participants had to decide between two given options that were simultaneously presented on a computer screen using the Psychophysics Toolbox (Brainard 1997) in MATLAB R2010a (MathWorks Inc., Natick, MA). A Bayesian adaptive algorithm was implemented so that parameter estimation is updated after each trial and is used for the calculation of the options in the next trial. This procedure provides the most informative offers near the individual’s indifference point (i.e. the point of indifference between two choice alternatives) and allows for a very efficient inference of decision-making parameters without post hoc parameter estimations. For delay and probability discounting tasks, a hyperbolic value function (Mazur 1987) was used describing that the subjective values of delayed (or probabilistic) reward decline hyperbolically according to the discounting rate *k*. For the mixed gambles task, we used a simple linear function in which loss aversion (*λ*) is the relative weighting of losses to gains in the participant’s decision (Tom et al. 2007). Individuals with higher impulsive decision-making are assumed to display higher *k* values in the delay discounting task, lower *k* values in probability discounting tasks, and lower *λ* values in the mixed gambles task.

### Data analyses

For all statistical analyses, BAYESMH and regress commands were used in Stata 15.0 (Stata Corp. 2017). The analyses applied Markov chain Monte Carlo (MCMC) simulation of posterior distributions (burn-in period = 5000, 100,000 simulations, thinning = two). In all analyses, we included baseline demographic characteristics (age, gender, IQ, income, and school graduation) as covariates in the regression analyses. The control variables were selected on the basis of theoretical assumptions and previous empirical evidence, rather than on the basis of statistical tests for group differences (difference tests might misleadingly suggest that there is no need to adjust the analyses for demographic characteristics, even if the groups differ considerably in these characteristics or associations of interest are biased by them). Finally, the BAYESTEST command was used to calculate the probabilities for the parameters to range in the pre-specified intervals. The primary data and the Stata do-files of the analyses can be downloaded from the Open Science Framework (OSF) under https://osf.io/ga68m/.

#### Addictive disorder groups at baseline

We analyzed 335 data sets because in three cases there were missing data in the decision-making tasks due to technical difficulties. To answer the first research question (group differences), we used Bayesian linear regression with the dummy-coded predictor SUD or ND group (control group as reference) and the logarithmic *k* or *λ* (for mixed gambles) values as outcomes. The comparison was based on our hypothesis that SUD or ND differ in their decision-making patterns from healthy controls. We had no global hypothesis regarding any group differences and no specific hypothesis regarding the difference between SUD and ND (exploratively compared in a post hoc analysis). We performed a regression analysis with dummy-coded groups instead of an ANOVA since the global F-test within ANOVA tests the full model against the null model, which in our case would only contain two intercepts (for three compared groups). Moreover, with the regression analyses with dummy-coded groups, other researchers investigating only one disorder group can later compare their results with ours.

As priors for the hypothesized regression coefficients, we used normal distributions with expectations (means) and variances as estimated in a previous study by Bernhardt et al. (2017); Fig. S1 in the supplemental material). This previous sample was an alcohol-dependent patient sample from a clinic (versus our community sample), was older (45 years versus 22 years in our sample), had a lower proportion of female participants in the SUD group (16% versus 53% in our sample), and had a higher SUD severity (median of 8 criteria versus 3 in our sample, Table S4). Since our overall aim was to get closer to the true value of group differences between individuals with and without AD, we assume that our priors were appropriately selected from an AD sample. However, these priors may have biased our results towards false-positive group differences. To counteract this problem, we use a range of priors as sensitivity analysis (see next subsection). The Bayesian analysis enabled us to calculate, according to the alternative hypotheses, how likely group differences were greater (for delay discounting) respectively lower than zero (for the other tasks)—given the data, the model, and the priors. In a post hoc analysis, the diagnostic groups were compared using the differences in the simulated posterior distributions of the SUD-control and the ND-control coefficients (using the implicit priors imposed for the comparison with controls). The percentages of values above and below zero were checked. Depending on the tasks and parameter interpretation, the percentage above zero indicates the extent of evidence that the SUD group has higher *k* values compared with the ND group (delay discounting), or that the SUD group has lower *k* and *λ* values compared with the ND group (probability discounting and loss aversion), respectively.

#### Relaxing the priors

We performed a ‘reverse-Bayes’ analysis to identify the most pessimistic prior assumption that still allows the conclusion in the hypothesized direction (Greenland 2006; Matthews 2001). We did this by starting from the current prior and reducing the expectation (mean) in 0.1 steps (leaving the variance unchanged).

#### Addictive disorder severity after one year

For the second research question (change model), we used Bayesian linear regression to analyze the relation between the logarithmic *k* or *λ* values and the differences between the number of AD criteria or QFI at follow-up minus baseline. We adjusted this comparison (difference of difference) for participants’ values at baseline (additional covariate) to prevent regression to the mean. Predictors and outcomes were both *z*-standardized, yielding standardized regression coefficients that have the same range as correlations. As priors, we expected the associations to be greater than zero for delay discounting respectively lower than zero for the other tasks. Specifically, we assumed a probability of 95% that the true association would range between 0 and 0.5 (respectively − 0.5 and 0), which corresponds to our assumption of medium associations between our laboratory tasks with later changes in diagnostic criteria or QFI. In a standard normal distribution, this assumption corresponds to an expectation of 0.25 (− 0.25) and a standard deviation of 0.127 (Fig. S2 in the supplemental material). For these priors, we did not perform sensitivity analyses, since our relatively broad prior (due to insufficient prior knowledge) will not strongly influence the posterior distribution. To find out whether the assumed predictive associations are specifically pronounced in the baseline disorder groups, we repeated the analyses with the same priors and additionally included an interaction term between groups (control group as reference group) and predictors (*k* or *λ*). High posterior probabilities for a positive (delay discounting) respective negative (probability discounting, loss aversion) interaction effect would support the conclusion that an association in the assumed direction was stronger within the baseline disorder groups than within the control group.

## Results

### Addictive disorder group comparisons at baseline

**Fig. 3:**
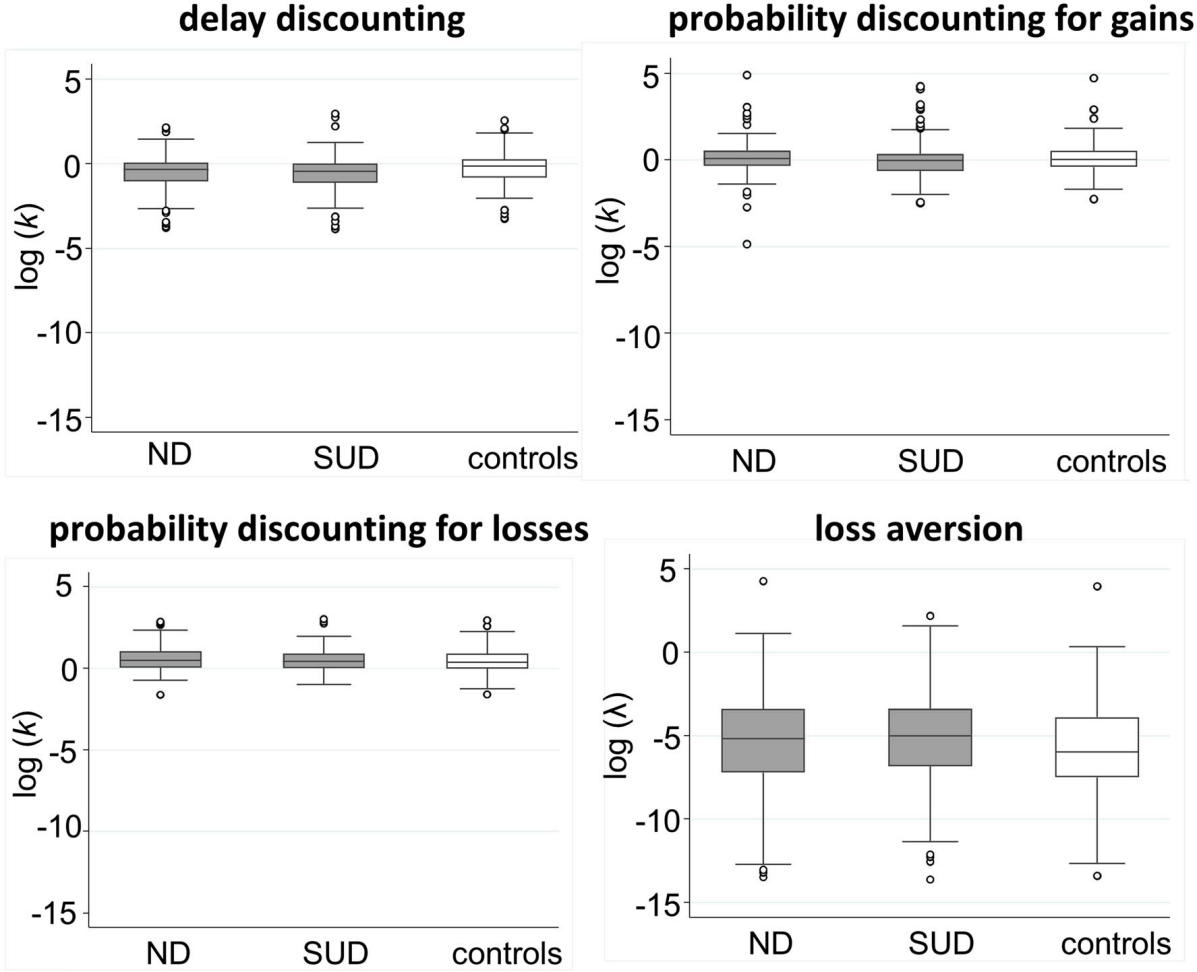
Box plots of the likelihood distributions corresponding to the four facets of impulsive decision-making and the three study groups: non-substance-related addictive disorders (ND), substance-related disorders (SUD), and the controls (in white)

In the next subsections, the posterior distributions of the Bayesian analyses are presented, which combine the assumed prior distributions and the likelihood (data) distributions shown in Fig. 3 (for details, see Table S5 and S6 in the supplemental material).

#### Delay discounting

Our analyses revealed a probability of 99% each that the SUD (alcohol, tobacco) group and the ND (Internet, gaming, gambling) group display steeper delay discounting than controls (Table 3), given the baseline data, the analytic model, and the priors (Fig. S1 in the supplemental material). The probability of the SUD group to have steeper delay discounting compared with the ND group was 73%. The evidence for the SUD-control comparison was very robust, i.e. not very sensitive to the prior assumption with an expectation of 0.37. The reverse-Bayes analysis showed that even with a prior with an expected value of − 0.10, group differences between SUD and control group can still be concluded with a 95% probability (Table S7 in the supplemental material). In contrast, the posterior distribution of the ND-control comparison was sensitive to the prior distribution. The reverse-Bayes analysis showed that the prior of the ND-control group difference must be at least 0.30 (medium) in order to still be able to conclude with a 95% probability that group differences exist.

**Table 3:** Results of the Bayesian linear regression analyses (with posterior, prior, and likelihood distributions) of the group differences in the decision-making parameters at baseline between the substance use disorder (SUD) group or the non-substance-related addictive disorder (ND) group and the control group (reference)

		Mean/beta against controls	95% credibility/confidence intervals	Probability (%) that the difference against controls is in hypothesized direction
Delay discounting log (*k*)				Log (*k*) difference > 0
SUD group	Posterior	0.30	0.06–0.54	99%
	Prior	0.37	0.10–0.64	
	Likelihood	0.24	− 0.02–0.51	
ND group	Posterior	0.21	0.03–0.40	99%
	Prior	0.37	0.10–0.64	
	Likelihood	0.08	− 0.16–0.34	
Probability discounting for gains log (*k*)				Log (*k*) difference < 0
SUD group	Posterior	− 0.13	− 0.38–0.12	84%
	Prior	− 0.16	− 0.44–0.13	
	Likelihood	− 0.09	− 0.37–0.19	
ND group	Posterior	− 0.11	− 0.30–0.08	88%
	Prior	− 0.16	− 0.44–0.13	
	Likelihood	− 0.06	− 0.33–0.21	
Probability discounting for losses log (*k*)				Log (*k*) difference < 0
SUD group	Posterior	− 0.26	− 0.49 to − 0.02	98%
	Prior	− 0.16	− 0.44–0.13	
	Likelihood	− 0.27	− 0.54 to − 0.01	
ND group	Posterior	− 0.19	− 0.38 to − 0.01	98%
	Prior	− 0.16	− 0.44–0.13	
	Likelihood	− 0.21	− 0.48–0.04	
Loss aversion log (*λ*)				Log (*λ*) difference < 0
SUD group	Posterior	− 0.05	− 0.29–0.19	65%
	Prior	− 0.44	− 0.71 to − 0.17	
	Likelihood	0.09	− 0.18–0.36	
ND group	Posterior	− 0.14	− 0.33–0.04	93%
	Prior	− 0.44	− 0.71 to − 0.17	
	Likelihood	0.12	− 0.14–0.37	

Baseline demographic characteristics (age, gender, IQ, income, and school graduation) were included as control variables in all analyses

#### Probability discounting for gains

We found a probability of 84% and 88% that the SUD group and the ND group, respectively, displayed lower probability discounting for gains (i.e. higher risk-seeking for gains) compared with the control group. The expected value of the prior was − 0.16, which corresponds to small group differences. The probability that the SUD group had lower probability discounting for gains than the ND group was 53%. In addition, a prior with an expected value of zero was applied to account for partly weak evidence for group differences in the literature. We found 77% and 65% evidence for lower probability discounting for gains in the SUD and ND group compared with the control group, respectively (Table S7 in the supplemental material).

#### Probability discounting for losses

The probabilities for lower probability discounting for losses (i.e. lower risk-seeking for losses) compared with the control group were 98% each for the SUD and the ND group, using a prior with an expected value of − 0.16 (small group differences). The probability that the SUD group had lower probability discounting for losses compared with the ND group was 66%. A prior with an expected value of zero (no group differences) still allowed the conclusion of a group difference with 95% probability for the SUD-control comparison, whereas for the ND-control comparison, the expected value of the prior had to be at least − 0.10 (Table S7 in the supplemental material).

#### Loss aversion

The probability that the SUD group had lower loss aversion compared with the control group was 65%, for the ND group this equalled 93%. The probability that the SUD group had lower loss aversion compared with the ND group was 27%. The posterior distribution of group comparisons was sensitive to the prior distribution (Fig. S1 in the supplemental material). Applying a prior with an expected value of zero, the results showed very weak evidence of 29% and 24% for lower loss aversion in the SUD and the ND group, respectively, compared with controls (Table S7 in the supplemental material).

In addition to the analyses presented above, we performed a sensitivity analysis concerning extreme values, i.e. we re-analyzed the data for the first research question by excluding participants outside the Stata box plot whiskers in Fig. 3 (i.e. values outside the lower quartile − 1.5 interquartile range (IQR) and the upper quartile + 1.5 IQR). The results do not differ from our original results, so we consider our results to be robust to the more extreme values of some participants (Table S8 in the supplemental material). Integrating all results, we found robust evidence for steeper delay discounting in SUD and for lower risk-seeking for losses in both SUD and ND compared with healthy controls.

### Addictive disorder severity after 1 year

#### Delay discounting

We found a probability of 99% that steeper delay discounting at baseline predicted an increase of SUD criteria after 1 year, for ND criteria this equalled 72% (Table 4). The credibility intervals for these associations were closely around 0 suggesting small effects. The group interaction analyses revealed probabilities of 85% (or 16%) for a stronger relation between steeper delay discounting and SUD (or ND) criteria change in the SUD (or ND) group compared with controls. The probabilities of a positive association between steeper delay discounting and increased substance- and non-substance-related QFIs 1 year later were 83% and 93%, respectively. The group interaction analyses revealed probabilities of 17% (or 80%) for a stronger relation between steeper delay discounting and SUD (or ND)-related QFI change in the SUD (or ND) group compared with controls. It suggests that the predictive associations between steeper delay discounting and symptom or QFI change were not particularly pronounced in one of the disorder groups.

**Table 4:** Results of the Bayesian linear regression analyses (with the posterior, prior, and likelihood distributions). Predictors were the logarithmic *k* or *λ* (for mixed gambles) values at baseline as indicators of impulsive decision-making. Outcomes were the differences between the addictive disorder severity of substance use disorder (SUD) or non-substance-related addictive disorder (ND) (number of fulfilled diagnostic criteria and quantity-frequency-index (QFI)) at 1-year follow-up minus baseline

		Mean/beta of association	95% credibility/confidence interval	Probability (%) that associations are hypothesized direction
Delay discounting log (*k*)				Association > 0
SUD criteria	Posterior	0.12	0.03–0.20	99
	Prior	0.25	0.00–0.50	
	Likelihood	0.10	0.01–0.20	
ND criteria	Posterior	0.03	− 0.06–0.11	72
	Prior	0.25	0.00–0.50	
	Likelihood	0.00	− 0.10–0.09	
QFI SUD-related	Posterior	0.04	− 0.05–0.15	83
	Prior	0.25	0.00–0.50	
	Likelihood	0.11	0.00–0.22	
QFI ND-related	Posterior	0.07	− 0.03–0.14	93
	Prior	0.25	0.00–0.50	
	Likelihood	0.05	− 0.04–0.15	
Probability discounting for gains log (*k*)				Association < 0
SUD criteria	Posterior	− 0.04	− 0.13–0.04	84
	Prior	− 0.25	− 0.50–0.00	
	Likelihood	− 0.02	− 0.11–0.08	
ND criteria	Posterior	− 0.06	− 0.14–0.03	90
	Prior	− 0.25	− 0.50–0.00	
	Likelihood	− 0.03	− 0.12–0.06	
QFI SUD-related	Posterior	0.02	− 0.09–0.12	40
	Prior	− 0.25	− 0.50–0.00	
	Likelihood	0.00	− 0.10–0.10	
QFI ND-related	Posterior	0.03	− 0.08–0.13	29
	Prior	− 0.25	− 0.50–0.00	
	Likelihood	0.03	− 0.06–0.12	
Probability discounting for losses log (*k*)				Association < 0
SUD criteria	Posterior	0.02	− 0.07–0.10	34
	Prior	− 0.25	− 0.50–0.00	
	Likelihood	0.05	− 0.04–0.14	
ND criteria	Posterior	− 0.09	− 0.17 to − 0.01	98
	Prior	− 0.25	− 0.50–0.00	
	Likelihood	− 0.06	−0.16–0.03	
QFI SUD-related	Posterior	0.03	− 0.07–0.14	26
	Prior	− 0.25	− 0.50–0.00	
	Likelihood	0.02	− 0.08–0.13	
QFI ND-related	Posterior	0.03	− 0.07–0.13	31
	Prior	− 0.25	− 0.50–0.00	
	Likelihood	0.02	− 0.08–0.11	
Loss aversion log (*λ*)				Association < 0
SUD criteria	Posterior	− 0.14	− 0.23 to − 0.06	99
	Prior	− 0.25	− 0.50–0.00	
	Likelihood	− 0.13	− 0.23 to − 0.03	
ND criteria	Posterior	− 0.10	− 0.18 to − 0.01	98
	Prior	− 0.25	− 0.50–0.00	
	Likelihood	− 0.07	− 0.17–0.03	
QFI SUD-related	Posterior	− 0.02	− 0.13–0.09	62
	Prior	− 0.25	− 0.50–0.00	
	Likelihood	− 0.04	− 0.16–0.07	
QFI ND-related	Posterior	− 0.02	− 0.10–0.09	57
	Prior	− 0.25	− 0.50–0.00	
	Likelihood	− 0.02	− 0.11–0.08	

Baseline demographic characteristics (age, gender, IQ, income, and school graduation) and participants’ baseline values in addictive disorder severity were included as control variables in all analyses

#### Probability discounting for gains

The posterior probabilities for negative relationships between probability discounting for gains (i.e. higher risk-seeking for gains) at baseline and increases of SUD and ND severity 1 year later were 84% and 90% for addictive criteria and 40% and 29% for QFIs, respectively. The group interaction analyses revealed low probabilities (39% and 40% for SUD and ND criteria, 39% and 46% for SUD- and ND-related QFIs, respectively) suggesting that these predictive associations were not particularly pronounced in one of the disorder groups.

#### Probability discounting for losses

The posterior probability for a negative relationship between lower probability discounting for losses at baseline and increased SUD criteria after 1 year was 34%. The probability for ND criteria was 98%. The credibility interval was closely around 0, indicating that the associations were weaker than a priori expected. We found probabilities of 26% and 31% that lower probability discounting for losses predicted an increase of substance- and non-substance-related QFIs, respectively. The group interaction analyses revealed low probabilities (15% and 19% for SUD and ND criteria, 7% and 23% for SUD- and ND-related QFI, respectively) suggesting that these predictive associations were not particularly pronounced in one of the disorder groups.

#### Loss aversion

The posterior probabilities for negative relationships between lower loss aversion at baseline and increased SUD and ND criteria 1 year later equalled 99% and 98%, respectively. While the credibility interval for the relationship between loss aversion and SUD criteria indicated a medium association, the interval for ND criteria ranged closely around zero. The group interaction analyses revealed probabilities of 93% (or 92%) for a stronger relation between steeper delay discounting and SUD (or ND) criteria change in the SUD (or ND) group compared with controls. For the substance- and non-substance-related QFIs, the probabilities were 62% and 57%, respectively, that lower loss aversion predicted higher quantity and frequency of use. The group interaction analyses revealed a probability of 52% for stronger associations within the SUD group, but this equalled 99% for stronger associations within the ND group. The association between lower loss aversion and non-substance-related QFI change was stronger negative in the ND group compared with the control group, where the association was even positive (− 0.14 for ND versus 0.22 for controls).

In sum, we found robust evidence that steeper delay discounting and lower loss aversion predicted increased SUD severity after 1 year while lower probability discounting for losses and lower loss aversion predicted increased ND severity. For loss aversion, the associations were specifically pronounced for the ND-specific QFI change within the ND group. In all analyses, associations were weaker than a priori expected (Fig. S2 in the supplemental material).

## Discussion

The results showed evidence for steeper delay discounting in SUD and lower probability discounting for losses in both SUD and ND compared with healthy controls at baseline, given the data, the analytic model, and the priors. Furthermore, we found that steeper delay discounting and lower loss aversion predicted increased SUD severity after 1 year while lower probability discounting for losses and lower loss aversion predicted increased ND severity.

As in previous studies (for an overview, see Amlung et al. 2017; Bickel et al. 2014; Kluwe-Schiavon et al. 2020), SUD was characterized by steeper delay discounting, which indicates impaired delay of gratification. This group difference was robust to prior assumptions. In contrast, the group difference between the ND and the control group was very sensitive to prior assumptions. For the comparison between ND and control group, one had to assume at least medium differences in order to be able to conclude ‘true’ group differences. However, medium group differences are not very likely when considering the evidence from a meta-analysis that showed small group differences in delay discounting of *r* = 0.16 between healthy controls and individuals with gambling disorder (Amlung et al. 2017). We therefore conclude that an overvaluation of immediate rewards and a lower ability to delay gratification may be particularly important in SUD, where immediate substance effects were chosen at the expense of long-term health benefits.

Moreover, we found evidence for lower probability discounting for losses in SUD and ND compared with healthy controls and weak evidence that both groups differ from each other. The results were robust to prior assumptions, i.e. they could be found even under the assumption of no or low group differences. The role of risk-seeking for losses in AD has so far only been addressed in few studies (Bernhardt et al. 2017; Ohmura et al. 2005; Takahashi et al. 2009). If one transfers the underlying prospect theory on outcome values and outcome probability weightings (for an overview, see Shead and Hodgins 2009) to an addiction-related example, lower risk-seeking for losses in AD may underlie the tendency to accept small but certain negative outcomes (e.g. having to go outside to smoke) instead of risking more uncertain negative outcomes whose probabilities are overestimated (e.g. withdrawal symptoms). Our results suggest that a lower risk-seeking for probabilistic losses could also be a candidate for a transdiagnostic process across different AD and that it should also be investigated in other mental disorders.

Against our hypothesis and previous findings from Bernhardt et al. (2017), we found weak evidence for lower probability discounting for gains and lower loss aversion in AD compared with healthy controls. The results were robust to prior assumptions. This suggests that AD is not characterized by an increased risk-taking propensity for uncertain rewards or a reduced sensitivity for potential losses. One explanation for the weak evidence for group differences in our study could be the sample composition. In the study from which our priors came, older AD patients were examined (Bernhardt et al. 2017). The neural value systems of this older patient sample may have been more strongly affected by aging processes (Denburg et al. 2005; Fein et al. 2007) and long-term sequelae of substance use (De Wit 2009), which may have led to greater group differences than in our young and mainly mildly affected community sample.

Concerning *predictive associations* between impulsive decision-making and AD, we found evidence that a steeper delay discounting predicts more SUD criteria after 1 year. This is in line with longitudinal studies that have found small to medium predictive associations between steeper delay discounting and an increased likelihood of smoking initiation (for an overview, see Barlow et al. 2017) and later alcohol involvement in healthy adolescents (Fernie et al. 2013). Consistent with our cross-sectional results at baseline, an inappropriate assignment of values to short-term rewards obtained through addictive behaviours at the expense of long-term health goals seems to be specifically important in the progression and relapse of AD involving the use of substances.

We found evidence that lower probability discounting for losses is more likely associated with the course of ND than the course of SUD. In other words, a lower risk-taking to avoid certain losses (e.g. accepting certain annoyances with the partner because of constant Facebook checking, than to risk to miss important messages) predicts more ND symptoms after 1 year but were not related to SUD symptom change. The missing relation to SUD symptom change is in line with a previous study that found low evidence for the association between probability discounting for gains and losses and alcohol use in healthy individuals (Bernhardt et al. 2017). The question arises why there is a specific predictive relationship to changes in ND symptoms. The probability discounting for losses paradigm combines participants’ attitude towards risk (e.g. overestimating the likelihood of an uncertain loss) and towards losses (e.g. reduced punishment sensitivity). Since lower loss aversion as indicator of reduced punishment sensitivity was also predictive for SUD symptom change, one could speculate that the first process is more important to answer the question: inter-individual differences in the tendency to overestimate the probability of uncertain losses may have a specific role in the course of AD without substance use. The specific role for ND may exist because negative consequences of behaviours such as gaming are much more uncertain compared with those of substance intake (e.g. intoxication).

Finally, we found evidence that lower loss aversion predicted increased AD criteria after 1 year. It indicates that people who are less sensitive to the possibility of losing compared with gaining something develop more addictive symptoms over time and have a steeper progression of symptoms. Lower loss aversion could result from increased reward sensitivity and lower punishment sensitivity that have both been demonstrated for AD (Beck et al. 2009; Dong et al. 2011; Volkow et al. 2010; Wrase et al. 2007). Interestingly, we found only weak evidence for group differences in loss aversion at baseline, and the baseline data distribution differed from the prior distribution derived from previous results from a 45-year-old patient sample (Bernhardt et al. 2017). Possibly, this could be explained by the young age of our sample. The continued structural and functional development of the prefrontal cortex into young adulthood underlies an increase of cognitive control abilities, e.g. the anticipation of negative consequences (Casey and Jones 2010). While immediate rewards may play an important role in the initial development of AD in adolescence, the weighting of rewarding effects against negative consequences may affect the course of AD severity only after complete neurobiological maturing in young adulthood.

Some of the impulsive decision-making facets predicted AD criteria changes. These associations were only small to medium, which could be due to the fact that changes in the number of DSM-5 criteria are count variables that only roughly reflect severity, i.e. they contain measurement errors that could lead to an underestimation of associations. Contrary to the AD criteria change, we found low evidence that any of the facets (despite loss aversion) predicted changes in quantity and frequency of use. This is in line with two meta-analyses that showed lower associations between QFI and delay discounting compared with the associations between AD criteria and delay discounting (Amlung et al. 2017). Impulsive decision-making could be a risk factor for AD symptoms, such as loss of self-control over time and resources spent on addictive behaviours or the devaluation of negative consequences, rather than a risk factor for substance use per se (MacKillop et al. 2011). The group-specific analyses revealed another interesting aspect. While we found strong evidence in the ND group that lower loss aversion predicted engagement in non-substance-related addictive behaviours, it was a higher loss aversion in the control group that predicted more behaviour. One explanation could be that we have included pathological and non-pathological use, where individuals with and without ND use the Internet or games for different reasons: Individuals with ND may use the Internet or games for positive reinforcement and overweight the gains compared with negative consequences. In contrast, individuals without ND may engage in Internet use or gaming for negative reinforcement, e.g. in order to distract from negative affect caused by higher punishment sensitivity in daily life. Further longitudinal studies comparing mechanisms of addictive behaviours with and without substance use are important to better understand their transition to risky use and AD.

The results of the study should be seen in the light of potential limitations. One strength of the study is that we recruited our sample of 19 to 27-year-old adults representatively from the community. However, this recruitment strategy may have led to a selection bias. We have a high proportion of individuals with mild and moderate addiction severity. Furthermore, at the time of our initial study planning, there were no established criteria available to diagnose ND (except for gambling disorder). To achieve homogenous group definitions, ND was diagnosed with modified DSM-5 SUD criteria with a lower threshold (two or more criteria) compared with those actually proposed for Internet gaming disorder in DSM-5 (five or more criteria). This lower addiction severity could also explain the observed lower group differences in our baseline data compared with our assumptions and is in line with previous studies that showed that AD severity might be linearly related to delay discounting (Alessi and Petry 2003). Another special characteristic of our sample is the higher proportion of females and of students (73%) amongst the respondents to our study invitation, although it should be noted that we recruited in a university city with a high proportion of students amongst the young adults. These specific characteristics of our sample may result in different courses of AD in our sample compared with the general population in the way that our participants may have a generally lower impulsive decision-making, a more shallow progression of AD severity (e.g. due to lower initial severity or gender differences; Nolen-Hoeksema 2004), or a higher probability of spontaneous remission after university graduation (Cousijn et al. 2018). In addition to selection biases, results may have been influenced by measurement errors in the laboratory tasks (e.g. low reliability) or in the self-reported measures of AD severity (e.g. underreporting). Both types of measurement errors would lead to an underestimation of true associations. Final sources of bias are unconsidered confounders (common causes), which underlie both impulsive decision-making and AD severity. Possible common causes could be neuroendophenotypes such as impulsivity (Robbins et al. 2012).

In a sample of young adults from the community with mild to moderate addiction severity, we found that impulsive decision-making characterizes AD and predicts the course of AD criteria but scarcely the engagement in addictive behaviours (QFI). Associations were evident in specific facets of impulsive decision-making and were mostly weaker than a priori expected. Impulsive decision-making is certainly only one of many intra-individual factors that influence the course of AD and has to be considered in interaction with other substance-related and social factors. Future studies are needed that apply longitudinal cross-lagged designs (see Fernie et al. 2013) and assess other putative risk factors (e.g. neuroendophenotypes, addictive behaviours of peers) to further understand the size of evidence and causal relationships between impulsive decision-making and AD. Different facets of impulsive decision-making should be measured as they seem to be differentially related to the types and course of AD. For such future analyses, our posterior distributions could be applied as new priors to facilitate cumulative evidence over time. We thereby propose to use Bayesian priors with larger variances to incorporate the heterogeneous results from studies with a different methodology and to be more certain whether results can be transferred to the entire population of individuals with AD.

## Electronic supplementary material

ESM 1 (PDF 778 kb)
