# Supplementary material for: Impulsive decision-making predicts the course of substance-related and addictive disorders
Source: Psychopharmacology (Berl). 2020 Jun 5;237(9):2709–24. doi: 10.1007/s00213-020-05567-z (PMC7501099; doi:10.1007/s00213-020-05567-z)
Supplement: Supplementary file 1 — (PDF 778 kb) [file 213_2020_5567_MOESM1_ESM.pdf]

Supplementary material for

Impulsive decision-making predicts the course of substance-related and addictive disorders

Anja Kräplin[1], Michael Höfler[1], Shakoor Pooseh[2,3], Max Wolff[1,2], Klaus-Martin Krönke[1], Thomas Goschke[1], Gerhard Bühringer[1,4,5], Michael N. Smolka[2]

[1] Faculty of Psychology, Technische Universität Dresden, Germany
[2] Department of Psychiatry, Technische Universität Dresden, Germany
[3] Freiburg Center for Data Analysis and Modeling, University of Freiburg, Germany
[4] IFT Institut für Therapieforschung, Munich, Germany
[5] Department of Clinical Research, Faculty of Health, University of Southern Denmark

Correspondence address

Anja Kräplin, Work group Addictive Behaviors, Risk Analysis and Risk Management, Faculty of Psychology, Technische Universität Dresden, Chemnitzer Straße 46, D-01187 Dresden, Germany. Email: anja.kraeplin@tu-dresden.de

Conflict of interest

Anja Kräplin, Michael Höfler, Shakoor Pooseh, Max Wolff, Klaus-Martin Krönke, Thomas Goschke, and Michael N. Smolka have no conflicts of interests to declare. Gerhard Bühringer has received unrestricted research grants from the Bavarian State Ministry of Finance (regulatory authority for and operator of the state gambling monopoly), via the Bavarian State Ministry of the Environment and Public Health, the German Federal Ministry of Economics and Technology (regulatory authority for the commercial gaming industry), and from public and private gambling providers.

Funding information

Open Access funding provided by Projekt DEAL. This research was supported by a grant from the Deutsche Forschungsgemeinschaft (DFG) for the Collaborative Research Centre 940 (SFB 940/1, SFB 940/2) and partly supported by grants from the DFG for the Collaborative Research Centre 265 (TRR 265) and the Research Unit 1617 (SM 80/7-1, SM 80/7-2).

Compliance with ethical standards

The study procedures were carried out in accordance with the Declaration of Helsinki. The Institutional Review Board at the Technische Universität Dresden approved the study (EK45022012). All participants were informed about the study and all provided written informed consent.

Methods

Recruitment and participants

Respondents and non-respondents

From 2013 to 2016, 18.000 inhabitants aged between 19 and 27 randomly taken from the registration office files of Dresden were invited by post to participate in our study and 1.856 responded to the invitation letter (10.3 %). We descriptively compared respondents and non-respondents regarding their birth year and gender. Respondents were fore likely female and more likely from the birth years 1992 to 1995 compared to 1988 to 1991 or 1996 (Table S1).

Table S1 Gender and birth years of respondents and non-respondents to our study invitation by post.

	**Respondents n (%)**	**Non-respondents n (%)**
**Gender**		
Female	1.077 (58.03%)	7.760 (48.07%)
**Birth year**		
1988	82 (4.4%)	668 (4.1%)
1989	70 (3.8%)	680 (4.2%)
1990	58 (3.1%)	692 (4.3%)
1991	55 (3.0%)	695 (4.3%)
1992	362 (19.5%)	2.638 (16.3%)
1993	360 (19.4%)	2.640 (16.4%)
1994	347 (18.7%)	2.652 (16.4%)
1995	319 (17.2%)	2.681 (16.6%)
1996	203 (10.9%)	2.797 (17.3%)

Sample size calculation

The sample size for the whole project was estimated using Stata 13 for multiple linear regression with power (1- β) = .80, significance level α= 0.05, and five covariates (age, gender, IQ, income, school graduation). We assumed moderate group differences at baseline with R^2=.05 according previous studies comparing individuals with gambling disorder, alcohol dependence, and Tourette syndrome on measures of executive functions and decision-making (Goudriaan et al. 2005; Goudriaan et al. 2006). The necessary sample size would have been N=235 in total. Furthermore, we assumed a drop-out rate of 30% during the first funding phase of the project (3 years). The final estimated sample size was 330 with 110 in each group.

Measurements

Addictive disorder groups at baseline

Table S2 Demographic characteristics of the sample at baseline and at one-year follow-up separately for the substance-related disorder (SUD) group, the non-substance-related addictive disorder (ND) group, and the control group.

**Baseline**	*SUD*	*ND*	*Controls*
n=338	100	118	120
	M (SD)	*M (SD)*	*M (SD)*
Age	21.8 (1.6)	21.8 (1.7)	21.9 (1.8)
Intelligence quotient	103.7 (8.9)	104.4 (10.1)	104.8 (10.4)
	n (%)	*n* (%)	*n* (%)
Female participants	53 (53.0 %)	70 (59.3 %)	76 (63.3 %)
Income < 1500 Euro per month	75 (75.8 %)	92 (77.0 %)	89 (75.4 %)
School graduation 'Abitur'[1)]	68 (68.7 %)	85 (72.0 %)	97 (82.2 %)
In education, pupils, or students	72 (72.7 %)	87 (73.7 %)	87 (73.7 %)[2)]
**One year follow-up**	*SUD*	*ND*	*CG*
n=312<br>*(92% from baseline)*	93 (93 %)	107 (91 %)	112 (93 %)
	M (SD)	*M (SD)*	*M (SD)*
Age	21.7 (1.7)	21.9 (1.7)	21.9 (1.8)
Intelligence quotient	103.7 (9.2)	104.3 (10.3)	105.1 (10.5)
	n (%)	*n* (%)	*n* (%)
Female participants	50 (53.8 %)	64 (59.8 %)	70 (62.5 %)
Income < 1500 Euro per month	70 (75.3 %)	83 (77.6 %)	85 (75.9 %)
School graduation 'Abitur'[1)]	64 (69.6 %)	76 (71.0 %)	92 (83.6 %)
In education, pupils, or students	68(73.9 %)	79 (73.8 %)	82(74.6 %)[2)]

Note: M = Means; SD = standard deviations

1) Abitur is the German school-leaving qualification required for university entrance.

2) Two participants refused to provide information.

Symptom severity according to groups

Table S3: Severity of substance-related and additive disorders (AD) according to the DSM-5 specifiers at baseline and follow-up (1 year later) separately for the substance use disorder (SUD) group, the non-substance-related addictive disorder (ND) group, and the control group.

Baseline AD severity according to DSM-5 specifiers			
	**SUD**<br>**n=100**	**ND**<br>**n=118**	**Controls**<br>**n=120**
DSM-5 SUD			
Tobacco-related	n=61	n=0	n=0
Mild	53%		
Moderate	33%		
Severe	14%		
Alcohol-related	n=55	n=0	n=0
Mild	84%		
Moderate	11%		
Severe	5%		
(Adapted) DSM-5 ND			
Internet-related	n=0	n= 101	n=0
Mild		61%	
Moderate		31%	
Severe		8%	
Gaming-related	n=0	n=34	n=0
Mild		44%	
Moderate		32%	
Severe		24%	
Gambling-related	n=0	n=1	n=0
Mild		100%	
Moderate		0%	
Severe		0%	
Shopping-related	n=0	n=0	n=0
Mild			
Moderate			
Severe			

Follow-up (1 year) AD severity according to DSM-5 specifiers

	**SUD** n=93	**ND** n=107	**CG** n=112
DSM-5 SUD			
Tobacco-related	n=37	n=2	n=1
Mild	51%	50%	0%
Moderate	41%	0%	100%
Severe	8%	50%	0%
Alcohol-related	n=24	n=10	n=11
Mild	87%	70%	73%
Moderate	13%	30%	27%
Severe	0%	0%	0%
(Adapted) DSM-5 ND			
Internet-related	n= 10	n= 53	n= 7
Mild	60%	66%	100%
Moderate	30%	19%	0%
Severe	10%	15%	0%
Gaming-related	n=0	n=10	n=1
Mild		60%	100%
Moderate		20%	0%
Severe		20%	0%
Gambling-related	n=0	n=1	n=0
Mild		100%	
Moderate		0%	
Severe		0%	
Shopping-related	n=0	n=1	n=2
Mild		100%	100%
Moderate		0%	0%
Severe		0%	0%

Note: multiple symptoms are possible for one person.

Data analyses

Sample for priors to test the first hypothesis (group differences)

Table S4 Demographic characteristics of the sample from a previous study (Bernhardt et al. 2017), which was used to estimate the prior distributions.

	Alcohol dependent patients	Healthy controls
**Number of participants**	114	98
	n (%)	*n* (%)
**Female participants**	18 (16.0 %)	17 (17.4 %)
	M (SD)	*M (SD)*
**Age**	44.8 (1.6)	43.8 (1.7)
	Median (range)	*Median (range)*
**Alcohol consumption in past year (g ethanol per drinking occasion)**	180 (36-810)	45 (0-135)
**Lifetime DSM-5 alcohol use disorder symptoms**	8 (3-11)	0 (0-7)

Note: M = Means; SD = standard deviations

Results

Table S5 Decision-making parameters separately for the substance use disorder (SUD) group, the non-substance-related addictive disorder (ND) group, and the control group.

**Decision-making facet**	**SUD**	**ND**	**Controls**
	M (SD)	M (SD)	M (SD)
**Delay discounting log(*k*)**	-5.12 (3.12)	-5.71 (3.27)	-5.80 (2.96)
**Probability discounting for gains log(*k*)**	-0.07 (1.24)	0.17 (1.10)	0.15 (0.95)
**Probability discounting for losses log(*k*)**	-0.57 (1.18)	-0.53 (1.30)	-0.27 (0.97)
**Loss aversion log(λ)**	0.55 (0.76)	0.59 (0.81)	0.47 (0.74)

Note: M = Means; SD = standard deviations

Table S6 Decision-making parameters separately for gender.

**Decision-making facet**	**Females**	**Males**
	M (SD)	M (SD)
**Delay discounting log(*k*)**	-5.36 (3.18)	-5.88 (3.03)
**Probability discounting for gains log(*k*)**	0.13 (1.16)	0.10 (0.97)
**Probability discounting for losses log(*k*)**	-0.55 (1.20)	-0.30 (1.10)
**Loss aversion log(λ)**	0.64 (0.76)	0.39 (0.77)

Note: M = Means; SD = standard deviations

Table S7

Results of the **reverse Bayesian analyses**[1), 2)] (with posterior, prior, and likelihood distributions) of the group differences in the decision-making parameters at baseline between the substance use disorder (SUD) group or the non-substance-related addictive disorder (ND) group and the control group.

		Mean/ Beta against controls	*95% credibility/ confidence interval*	*Probability (%) that the difference against controls is in hypothesized direction*
Delay discounting log(*k*)[1)]				log(*k*) difference >0
SUD group	*Posterior*	0.20	-0.02−0.43	96%
	Most pessimistic prior	-0.10	-0.37−0.20	
	Likelihood	0.24	-0.02− 0.51	
ND group	*Posterior*	0.04	-0.01−0.35	97%
	Most pessimistic prior	0.30	0.07−0.60	
	Likelihood	0.08	-0.16− 0.34	
Probability discounting for gains log(*k*)[2)]				log(*k*) difference <0
SUD group	*Posterior*	-0.09	-0.33− 0.15	77%
	Zero prior	0.00	-0.28−0.29	
	Likelihood	-0.09	-0.37−0.19	
ND group	*Posterior*	-0.04	-0.22− 0.15	65%
	Zero prior	0.00	-0.28−0.29	
	Likelihood	-0.06	-0.33−0.21	
Probability discounting for losses log(*k*)[1)]				log(*k*) difference <0
SUD group	*Posterior*	-0.25	-0.36− -0.04	99%
	Most pessimistic prior	0.00	-0.28−0.29	
	Likelihood	-0.27	-0.54− -0.01	
ND group	*Posterior*	-0.17	-0.35− 0.02	96%
	Most pessimistic prior	-0.10	-0.38−0.19	
	Likelihood	-0.21	-0.48−0.04	
Mixed gambles log(λ)[2)]				log(λ) difference <0
SUD group	*Posterior*	0.07	-0.18−0.31	24%
	Zero prior	0.00	-0.27− 0.61	
	Likelihood	0.09	-0.18−0.36	
ND group	*Posterior*	0.07	-0.12−0.25	29%
	Zero prior	0.00	-0.27− 0.61	
	Likelihood	0.12	-0.14−0.37	

Note: Baseline demographic characteristics (age, gender, IQ, income, and school graduation) were included as control variables in all analyses.
1) For the two facets of impulsive decision-making, where we have drawn conclusions for group differences (delay discounting, probability discounting for losses), we have performed a "reverse-Bayes" analysis. Starting from the current prior used for the paper, we reduced the expected value in 0.1 steps until we identified the most pessimistic prior expectation that still allows the conclusion with a probability of 95% (leaving the variance unchanged).
2) For the two facets of impulsive decision-making where we did not draw conclusions for group differences, but the literature suggests weak evidence for group differences (probability discounting for gains, loss aversion), we used a prior with an expected value of zero (leaving the variance unchanged).

Table S8

Results of the Bayesian linear regression analyses (with posterior, prior, and likelihood distributions) of the group differences in the decision-making parameters at baseline between the substance use disorder (SUD) group or the non-substance-related addictive disorder (ND) group and the control group as reference group. Results are **without extreme values** according to Stata box plot (values outside the lower quartile - 1.5 inter quartile range (IQR) and the upper quartile + 1.5 IQR).

		Mean/ Beta against controls	*95% credibility/ confidence interval*	*Probability (%) that the difference from controls is in hypothesized direction*
Delay discounting log(*k*)				log(*k*) difference >0
Controls *n*= 5 excluded				
SUD group (*n*=6 excluded)	*Posterior*	0.35	0.013−0.56	99%
	Prior	0.37	0.10−0.64	
	Likelihood	0.24	-0.02− 0.51	
ND group (*n*=8 excluded)	*Posterior*	0.21	0.04−0.38	99%
	Prior	0.37	0.10−0.64	
	Likelihood	0.08	-0.16− 0.34	
Probability discounting for gains log(*k*)				log(*k*) difference <0
Controls *n*= 8 excluded				
SUD group (*n*=15 excluded)	*Posterior*	-0.27	-0.42− 0.12	99%
	Prior	-0.16	-0.44−0.13	
	Likelihood	-0.09	-0.37−0.19	
ND group (*n*=12 excluded)	*Posterior*	-0.03	-0.17− 0.10	69%
	Prior	-0.16	-0.44−0.13	
	Likelihood	-0.06	-0.33−0.21	
Probability discounting for losses log(*k*)				log(*k*) difference <0
Controls *n*= 8 excluded				
SUD group (*n*=10 excluded)	*Posterior*	-0.24	-0.41− -0.08	99%
	Prior	-0.16	-0.44−0.13	
	Likelihood	-0.27	-0.54− -0.01	
ND group (*n*=15 excluded)	*Posterior*	-0.17	-0.31− -0.02	99%
	Prior	-0.16	-0.44−0.13	
	Likelihood	-0.21	-0.48−0.04	
Mixed gambles log(λ)				log(λ) difference <0
Controls *n*= 4 excluded				
SUD group (*n*=7 excluded)	*Posterior*	-0.05	-0.29−0.19	65%
	Prior	-0.44	-0.71− -0.17	
	Likelihood	0.09	-0.18−0.36	
ND group (*n*=6 excluded)	*Posterior*	-0.14	-0.33−0.04	93%
	Prior	-0.44	-0.71− -0.17	
	Likelihood	0.12	-0.14−0.37	

Note: Baseline demographic characteristics (age, gender, IQ, income, and school graduation) were included as control variables in all analyses.

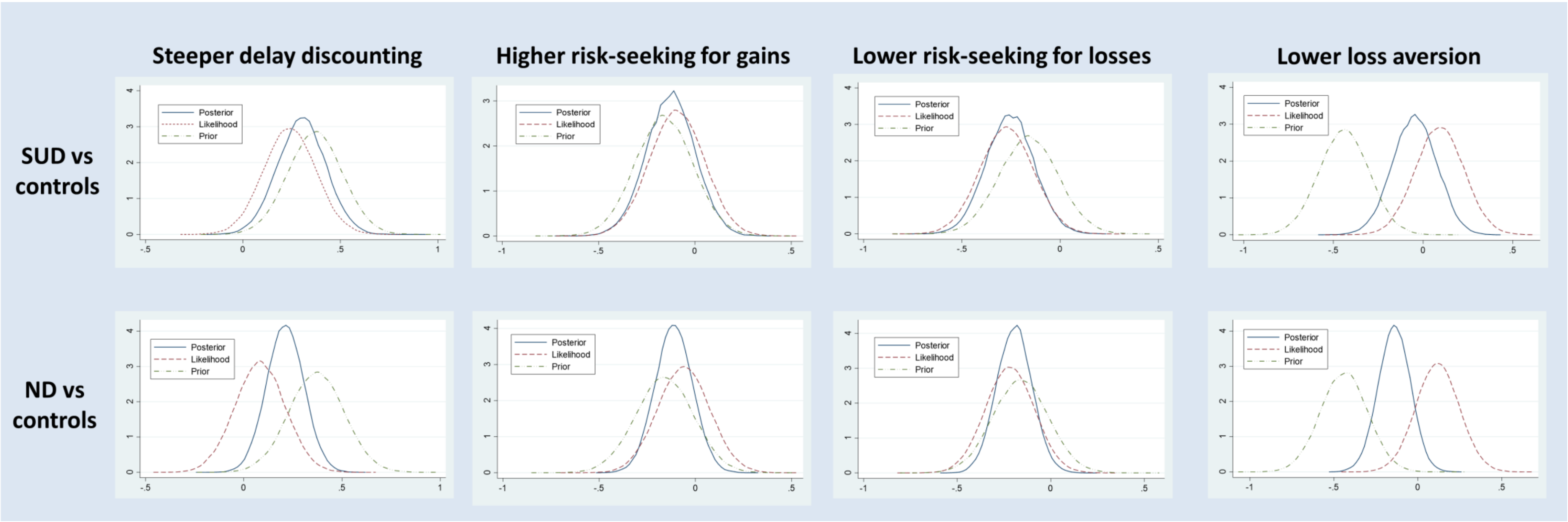

Fig. S1. Prior, likelihood, and posterior distributions of the Bayesian linear regression analyses to answer the first research question (group differences). Predictors were the dummy-coded groups (substance use disorder (SUD) group or non-substance-related addictive disorders (ND) group, control group as reference). Outcomes were the logarithmic *k* or λ (for mixed gambles) values as indicators of impulsive decision-making. Positive group differences (x axis) indicate that the SUD or ND group had higher values compared to the controls group. We hypothesized that steeper delay discounting (group difference > 0), lower probability discounting for gains, lower probability discounting for losses, and decreased loss aversion (group differences < 0 each) characterize individuals with SUD and ND compared to healthy controls. As priors for regression coefficients we used normal distributions with expectations and variances as estimated in a previous study from our lab (Bernhardt et al. 2017).

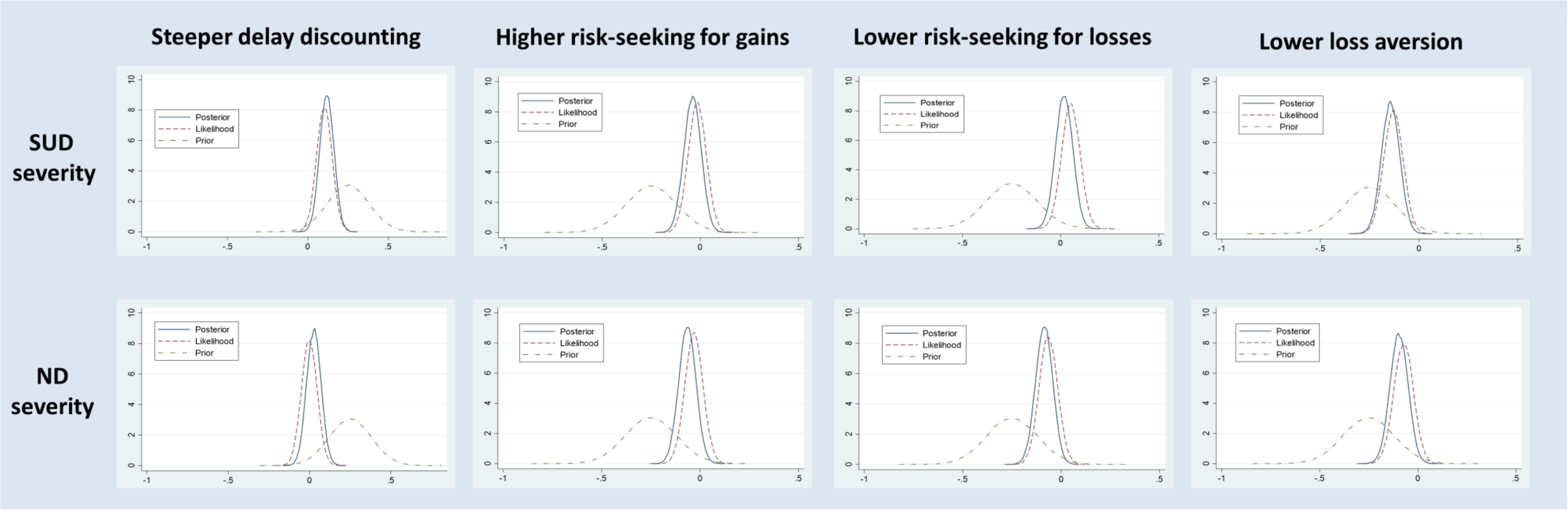

Fig. S2. Prior, likelihood, and posterior distributions of the Bayesian linear regression to answer the second research question (change model). Predictors were the logarithmic *k* or λ (for mixed gambles) values as indicators of impulsive decision-making. Outcomes were the differences between substance use disorder (SUD) or non-substance-related addictive disorder (ND) severity (number of fulfilled criteria) at follow-up minus baseline. Predictors and outcomes were both z-standardized, yielding standardized regression coefficients that have the same range as correlations (x axis). We hypothesized that steeper delay discounting (correlation > 0), lower probability discounting for gains, lower probability discounting for losses, and lower loss aversion (correlations < 0 each) predict increased addictive disorder severity, i.e. an increased number of fulfilled criteria. Concerning the priors, we assumed a probability of 95% that the true association would range between 0 and 0.5 (resp. -0.5 and 0) which corresponds with an expectation of 0.25 (-0.25) and a standard deviation of 0.127 in a normal distribution.

Supplemental references

Bernhardt N et al. (2017) Impulsive decision making in young adult social drinkers and detoxified alcohol-dependent patients: a cross-sectional and longitudinal study. Alcoholism: Clinical and Experimental Research 41:1794-1807. https://doi.org/10.1111/acer.13481

Goudriaan AE, Oosterlaan J, de Beurs E, van den Brink W (2005) Decision making in pathological gambling: a comparison between pathological gamblers, alcohol dependents, persons with Tourette syndrome, and normal controls. Cognitive Brain Research 23:137-151. https://doi.org/10.1016/j.cogbrainres.2005.01.017

Goudriaan AE, Oosterlaan J, de Beurs E, van den Brink W (2006) Neurocognitive functions in pathological gambling: a comparison with alcohol dependence, Tourette syndrome and normal controls. Addiction 101:534-547. https://doi.org/10.1111/j.1360-0443.2006.01380.x
